# Dataset of the savings rate and future wealth variables for a sample of Chilean households

**DOI:** 10.1016/j.dib.2022.108294

**Published:** 2022-05-17

**Authors:** Carlos Madeira

**Affiliations:** Central Bank of Chile, Santiago, Chile

**Keywords:** Pensions, Wealth, Social security system, Savings rates, Earnings risk

## Abstract

This article provides a pooled cross-sectional sample of Chilean households from 4 survey waves (1997, 2007, 2012, 2017). The data has information on the demographics of the household, labor participation and occupation, savings rates, plus wealth of different sources. The data is available in both Excel and Stata formats. It is an important data for the study of savings, wages, pensions and wealth inequality.

## Specifications Table

**Table:** 

Subject	Economics, Econometrics and Finance.
Specific subject area	Household Finance. Pensions. Social security. Labor Economics.
Type of data	Table (Excel format and Stata dta files)
How data were acquired	Data combines publicly available raw data from the Chilean Family Expenditures Survey (EPF, 1997, 2007, 2012, 2017 waves) and the Chilean Employment Survey (NENE, 1996-2016 waves) with a life cycle model of savings to create several measures of the savings rate and expected lifetime wealth. Hardware: data analysis was performed in a standard notebook with an Intel Core i7-4700HQ 2.40GHz processor with 16.0 GB of RAM. Software: Stata MP-6 (version 15.1).
Data format	Analyzed
Description of data collection	Data consists of demographics (gender, age, education, children and older household members), labor market information (occupational unemployment rate, labor income volatility), savings rates, and expected wealth components (decomposed into contributory pension, public pension, labor earnings, plus non-labor sources such as rents, transfers and financial assets income).
Data source location	Institution: Instituto Nacional de Estadísticas (INE, in English, Bureau of Official Statistics) City/Town/Region: Santiago Country: Chile https://www.ine.cl/estadisticas/sociales/ingresos-y-gastos/encuesta-de-presupuestos-familiares (EPF) https://www.ine.cl/estadisticas/sociales/mercado-laboral/ocupacion-y-desocupacion (NENE)
Data accessibility	With the article. Repository name: Mendeley Data Data identification number (DOI): 10.17632/dyp8yr2sr2.1. Direct URL to data: https://data.mendeley.com/datasets/dyp8yr2sr2/1
Related research article	C. Madeira, The impact of the Chilean pension withdrawals during the Covid pandemic on the future savings rate, *J Int Money Finance*, 126 (2022) 102650.

## Value of the Data

• The data can be used to study the distribution of pension wealth in Chile and its adequacy to support future consumption (Madeira [1], OECD [7]).
• The data also can be used to analyse the households’ savings rate and its heterogeneity across different groups (Gandelman [3]), which is particularly important due to the low savings in Latin America relative to other continents (Cavallo and Serebrisky [2]).
• The data can be used to study wealth inequality in Chile and how human capital wealth in the form of future wages, government transfers and pension benefits attenuate the unequal distribution of financial wealth (Madeira [1], Piketty [6]).

## 1 Data

One data file is included in both Excel and Stata formats: SRates_Dib.xlsx (SRates_Dib.dta). The dataset consists of demographics (gender, age, education, children and older household members), labor market information (occupational unemployment rate, labor income volatility), savings rates, and expected wealth components (decomposed into contributory pension, public pension, labor earnings, plus non-labor sources such as rents, transfers and financial assets income). The dataset includes 33,538 households from the 1997, 2007, 2012 and 2017 waves of the Chilean Family Expenditures Survey. The variables include Household identifier variables and population weights, Demographic variables (gender, age, education, spouse occupation, couple, child and senior persons), Work and income variables, Savings rates and consumption flows variables, Ratios of household wealth as a fraction of permanent household income, Betas for the linear correlation between unemployment risk and income volatility of the different 538 worker types with the aggregate consumption kernel pricing returns and the pension fund returns.

This is the list of variables available in the dataset:

Household identifier variables and population weights –
hogar “household identifier of each EPF wave”
folio_hogar “household identifier for the pooled cross-section of all the EPF waves”
year “Year of the EPF Survey wave”
factor_all “expansion factor (population weight) of the household in the survey”
id “group cluster identifier”
Demographic variables (gender, age, education, spouse occupation, couple, child and senior persons) –
sexo “Gender of the household head (1 Male, 2 Female)”
edad “age (in years) of the household head”
educ “education: elementary, secondary, university”
educ_ecf “Education level of the respondent (only 2017 wave)”, with values 1 “Elementary education” 2 “Secondary education” 3 “Technical or Some college” 4 “College education” 5 “Post-graduate education”
ocup_female_spouse “female partner of the household is employed”
couple_d “household has a couple among its members”
d_child “dummy for whether the household has a child”
num_sen “dummy for whether the household has a senior citizen (above age 65) among its members”
Work and income variables –
ILFP “dummy for whether the main income of the household comes from informal employment”
dummy_region “dummy for whether the household lives in regions outside of the Metropolitan Capital region”
quintile_h “household national income quintile”
ytoth “log of the total household permanent income (monthly)”
sd_ln_inc_sect “annual standard deviation of the household labor income”
unemp_sect “unemployment risk of the household”
Savings rates and consumption flows variables –
CBeta “fraction of wealth that should be consumed each year in a standard life cycle model”
SRate “ratio of the current saving rate in terms of the permanent income”
SRatePI “ratio of the permanent saving rate in terms of the permanent income”
aggSRate “ratio of the total current saving rate in terms of the permanent income”
aggSRatePI “ratio of the total permanent saving rate in terms of the permanent income”
Ratios of household wealth as a fraction of permanent household income –
Rytoth_c “Household income surprise”
R_TotalWI_hh “Discounted total wealth”
R_PW2I_hh “Discounted total pension wealth”
R_FE_hh “Discounted labor earnings wealth”
R_PW2I_hh_NoSy “Discounted contributory pension wealth”
R_PW2I_APS “Discounted solidarity pension wealth”
R_PWI_hh_past “Discounted current contributory pension wealth”
R_PWI_hh_NoSy “Discounted contributory pension wealth”
R_FENL_hh “Discounted non labor earnings wealth”
R_FErent “Discounted rent wealth”
R_FEtransfers “Discounted transfers wealth”
R_FEfinassets “Discounted financial income wealth”
Betas for the linear correlation between unemployment risk and income volatility of the different 538 worker types with the aggregate consumption kernel pricing returns and the pension fund returns –
BetaPF_unemployed “Beta between the occupational unemployment with the Pension Fund real rate of return”
BetaPF_sd_ln_ing_tot_ocup3 “Beta between the occupational income volatility with the Pension Fund real rate of return”
Beta_unemployed “Beta between the occupational unemployment with the Consumption Pricing Kernel real rate of return”
Beta_sd_ln_ing_tot_ocup3 “Beta between the occupational income volatility with the Consumption Pricing Kernel real rate of return”

## 2 Experimental Design, Materials and Methods

The data consists of demographics, labor earnings and risk (occupational unemployment rate, labor income volatility) and a simulation of the future contributory pension wealth plus public solidarity benefits for a sample of Chilean households (Madeira [1]). The model calibration accounts for the life-cycle optimization problem of the households to smooth their income and wealth within a framework of no uncertainty and no credit constraints (similar to previous literature, see the references in Madeira [1]).

The data uses the pooled cross-section sample of households from the Chilean Family Expenditures Survey (in Spanish, *Encuesta de Presupuestos Familiares*, hence on EPF) between 1997 until 2017. The dynamics of labor force participation, formal versus informal work and unemployment are calibrated from the Chilean Employment Survey (in Spanish, *Encuesta Nacional de Empleo*, hence on NENE), according to 538 workers’ types which are obtained from the multivariate vector of the workers’ sex, age, education, industry and region (Madeira [4]). Furthermore, the dataset includes the heterogeneous covariance between the workers types’ unemployment rate and labor earnings volatility (Madeira [4]) with the aggregate pension funds returns and the stochastic discount rate returns obtained from a consumption pricing kernel (Madeira [1]).

The wealth variables are reported as a ratio of the permanent income of the household and multiplied by a discounted smooth consumption factor to be measured in terms of an annual flow of the future expected wealth, which would be consumed by a rational agent with no financial frictions (Madeira [1]).

Users can download (at no cost) the raw data of all the EPF and ENE surveys from the website of the Chilean Institute of National Statistics.

ENE: https://www.ine.cl/estadisticas/sociales/mercado-laboral/ocupacion-y-desocupacion.

EPF:https://www.ine.cl/estadisticas/sociales/ingresos-y-gastos/encuesta-de-presupuestos-familiares.

The applied model that was calibrated from the raw data is explained in detail in the online file “Methodology.pdf”. The codes used to create the variables are explained in detail in the file README_JIMF_Codes_Summary.docx and CODES_JIMF.zip includes all the 45 Stata software codes used in the article. These files are publicly available with the data in the repository Mendeley Data.

The online file in Mendeley Data CODES_JIMF.zip includes all the software codes with detailed comments on the methods used inside each code. Here I provide a brief summary of those codes. The “M_EPF_analysis.do” do file replicates the analysis of the article, by calling all the algorithms and doing each code in sequenced steps until all the data formatting and analysis is completed.

The codes pctile_wgts.do, mean_wgts.do, and linear_reg_impute3.do create conditional group percentiles, mean values and imputations for missing values in the micro survey data.

A second set of codes formats the Income and Employment Survey creating unemployment risk and income volatility statistics for 538 worker types for the period 1990 until 2017, with worker types given by gender, education, region, industry of occupation, age, income quintile. These codes include: esi_format.do (formats the cross-sectional waves), panel_esi_allyrs_FLP.do (formats rotating samples between 2 years for the ESI workers in the labor force), panel_esi_ILFP.do (formats rotating samples between 2 years for the ESI household members outside of the formal labor force), panel_esi_income_growth0.do (calculates the real income growth of the worker types), layoff_jobfind0.do (calculates the separation rate and job finding rate of the worker types), income_shock0.do (creates the labor income volatility for all household members), p_income.do (calculates the permanent labor income for the workers), Consumption_WageVolatility.do (creates the consumption kernel returns and the pension fund returns and its beta values with the unemployment risk and income volatility of the 538 worker types, see Table 1 in the research article).

**Table 1:** Description of the dataset (SRates_Dib.xlsx, plus its Stata .dta version) provided in this article.

Analyzed dataset	Description
*SRates_Dib.xlsx (SRates_Dib.dta)*	*Demographics, savings rates and calibrated wealth ratios of the EPF households.*

A third set of codes formats the Chilean Expenditure Survey waves with similar variables for every year (1997, 2007, 2012, 2017): EPF_2017.do, EPF_2017_DurSDurNDur_Tot.do, format_epf_1997.do, format_epf_2007.do, format_epf_2012.do, format_epf_2017.do // It formats the EPF 2017 data with the same variables and formats of other years. The code format_epf_all.do joins all the EPF waves.

A fourth set of codes joins the EPF data with the Employment and Income Survey worker type statistics for each of the 538 worker types across survey waves. The codes then estimate past and expected future pension contributions for each worker. This set of codes includes: EPF_labor_risk_vintage.do, EPF_all_LFP_ILFP_FE_PW_PWpast.do, income_potential.do, import_FE_PW_PW_past.do, generate_FE_FENL_PW_PWpast_TW_hh.do, generate_log_Wealth.do, Pension_tope_income.do.

A fifth set of codes calibrates the pension system parameters for Chile in previous years, the pension withdrawals (August 2020, December of 2020, April 2021), the current policy reforms in 2022, and the counterfactual scenarios for the future reforms. This set of codes includes: Ingreso_bruto.do, Pension_income.do, Pension_PBS.do, Pension_PGU.do, Pension_PBS_2019.do, Pension_PBS_2008.do, Pension_PBS_PASIS.do, Pension_PASIS.do, Pension_Contr_APS_total.do, Pension_Reparto.do, Pension_Future.do, Retiro_AFP.do, PensionReformsFormat.do, predictLS_old_new1.do.

The sixth set of codes analyses the data and providing the results in Madeira [1]: Regs_analysis_tot.do (this code creates Table 3 and Table 4 in Madeira [1]; it also creates Tables A.1, A.2, A.5, A.6, A.7, A.8, A.9, A.10, A.11, A.12, A.13, A.14 in the appendix of Madeira [1]), Tables_Figures_RepRatios.do (this code creates Table 8 plus Fig. 1 and Fig. 2 in Madeira [1]; it also creates Table B.4 in the appendix of Madeira [1]), Tables_SRates.do (this code creates Table 6 and Table 7 in Madeira [1]; it also creates Table A.3 and Table A.4 in the appendix of Madeira [1]; it also creates Tables B.2 and B.3 in the appendix of Madeira [1]), Tables_X.do (this code creates the descriptive Table 2 in Madeira [1]).

All the methods (in Stata do-files), theoretical methodology, and the datasets are published online with the Mendeley Data (Madeira [5]): https://data.mendeley.com/datasets/dyp8yr2sr2/1.

## CRediT authorship contribution statement

**Carlos Madeira:** Conceptualization, Methodology, Software, Validation, Formal analysis, Investigation, Resources, Data curation, Writing – original draft, Writing – review & editing, Visualization, Supervision, Project administration, Funding acquisition.

## Declaration of Competing Interest

The author declares that he has no known competing financial interests or personal relationships which have or could be perceived to have influenced the work reported in this article. I received no funding from any institution besides my employer which is the Central Bank of Chile. Furthermore, there are no patents or impediments to publication, including the timing of publication, with respect to the intellectual property of the article or the associated dataset.

## Data Availability

Calibrated wealth ratios and labor-demographic variables across the 1997-2017 waves of the Chilean Family Expenditures Survey (Original data) (Mendeley Data).
